# CAREER PERCEPTIONS AND IDEALS FOR CLINICALLY FOCUSED AND ACADEMIC PSYCHOLOGY CAREERS: A MIXED-METHODS STUDY

**DOI:** 10.1093/geroni/igz038.037

**Published:** 2019-11-08

**Authors:** Jessica Strong, Rebecca Allen, Caitlan Tighe, Mary Jacobs, Hillary Dorman, Benjamin Mast

**Affiliations:** 1 VA Boston Healthcare System, Boston, Massachusetts, United States; 2 University of Alabama, Tuscaloosa, Alabama, United States; 3 University of Louisville, Louisville, Kentucky, United States

## Abstract

More psychologists who have specialty training in geriatrics are needed to meet the growing demand. However, there is a shortage of individuals in academic geropsychology, which feeds the clinical geropsychology pipeline. Barriers to recruiting trainees into jobs in academia are not well understood. The current mixed-methods study examined trainees’ perceptions of clinically-focused and academic jobs, and discrepancies between professional psychologists actual and ideal job activities. Results found that trainees have less accurate perceptions of the activities of academic compared to clinically-focused jobs. Interviews with trainees revealed negative perceptions of the university system, including bureaucracy, salary, and perceived workload. However, professional psychologists, both clinical and academic, reported high agreement between actual and ideal activities. Academic psychologists reported desiring more time in clinical work, without reducing research or teaching time. Clinically-focused psychologists desired and increase in research and teaching time without sacrificing clinical activities. Each group discussed struggles in obtaining work-life balance.

